# Chloroquine-induced QTc prolongation in COVID-19 patients

**DOI:** 10.1007/s12471-020-01429-7

**Published:** 2020-04-29

**Authors:** M. P. H. van den Broek, J. E. Möhlmann, B. G. S. Abeln, M. Liebregts, V. F. van Dijk, E. M. W. van de Garde

**Affiliations:** 1 Department of Clinical Pharmacy, St. Antonius Hospital, Utrecht/Nieuwegein, The Netherlands; 2 Department of Cardiology, St. Antonius Hospital, Nieuwegein, The Netherlands; 3 Division of Pharmacoepidemiology and Clinical Pharmacology, Department of Pharmaceutical Sciences, Utrecht University, Utrecht, The Netherlands

**Keywords:** COVID-19, Coronavirus, SARS-CoV‑2, Chloroquine, QT prolongation, Electrocardiogram

## Abstract

**Background:**

In the battle against the SARS-CoV‑2 pandemic, chloroquine has emerged as a new potential therapeutic option for the treatment of infected patients. A safety consideration for the application of chloroquine is its QTc-prolonging potential. Thus far, no data are available on the QTc-prolonging potential of chloroquine in COVID-19 patients.

**Objective:**

To assess the degree of chloroquine-induced QTc prolongation in hospitalised COVID-19 patients.

**Methods:**

A baseline electrocardiogram (ECG) and ECGs recorded during chloroquine treatment were retrospectively collected in patients suspected of having COVID-19. The QTc interval was calculated by computerised and manual interpretation. Baseline and follow-up QTc intervals were compared using the paired samples *t*-test.

**Results:**

A total of 95 patients had a baseline ECG recording and at least one ECG recording during chloroquine therapy. Chloroquine treatment resulted in a mean QTc prolongation of 35 ms (95% CI 28–43 ms) using computerised interpretation and 34 ms (95% CI 25–43 ms) using manual interpretation. No torsade de pointes was observed during chloroquine treatment. After manual review, 22 patients (23%) had a QTc interval exceeding 500 ms during chloroquine treatment. None of these patients had a prolonged QTc interval prior to the initiation of chloroquine treatment.

**Conclusions:**

Chloroquine significantly prolongs the QTc interval in a clinically relevant matter. This highlights the need for ECG monitoring when prescribing chloroquine to COVID-19 patients.

## What’s new?

- Chloroquine prolongs the QTc interval in COVID-19 patients in a significant and clinically relevant matter.
- It is recommended that QTc intervals be monitored by recording a baseline electrocardiogram (ECG) and then a further ECG during chloroquine treatment.

## Introduction

In the battle against the SARS-CoV‑2 (COVID-19) pandemic, chloroquine has emerged as a new potential therapeutic option for the treatment of infected patients [1]. This has led to widespread use of this drug for COVID-19 in many countries. Chloroquine belongs to the class of quinoline antimalarials.

A common side effect of chloroquine is prolongation of the corrected QT (QTc) interval. Several studies have shown that chloroquine prolongs the QTc interval in humans in a dose- and concentration-dependent manner [2–4]. Chloroquine blocks the rapidly activating delayed rectifier K^+^ current, encoded by the human-ether-a-go-go-related gene (hERG). Chloroquine has been shown to inhibit the hERG K^+^ channels in a concentration- and time-dependent manner [5]. Inhibition of the hERG channel can lead to prolongation of the action potential duration and, consequently, of the QT interval of the electrocardiogram (ECG), which may lead to torsade de pointes, a polymorphic ventricular tachyarrhythmia [6]. Chloroquine and hydroxychloroquine are on the CredibleMeds list of drugs ‘known to cause torsade de pointes’.

As yet, no data are available on QTc prolongation by chloroquine in COVID-19 patients. These data are relevant because a different dosing scheme is used for chloroquine in COVID-19 patients, and the effect of the disease itself on QTc intervals is unknown. Therefore, the objective of our study is to identify the QTc-prolonging potential of chloroquine in hospitalised COVID-19 patients.

## Methods

A retrospective, observational cohort study was conducted at a large non-university teaching hospital (St. Antonius Hospital, Utrecht/Nieuwegein, The Netherlands). All patients were aged 18 years or older, suspected of having COVID-19 disease and were hospitalised for that reason between 8 and 27 March 2020. The study protocol was reviewed by the MEC‑U Medical Ethics Committee (W20.065). The need for written informed consent to study clinical data was waived because of the urgency of the pandemic.

According to the Dutch Working Party on Antibiotic Policy (SWAB) chloroquine and hydroxychloroquine can be considered as a treatment option for COVID-19 patients that are admitted to the hospital and require respiratory support [7]. Chloroquine was chosen for availability reasons. The dosage regimen for chloroquine was a loading dose of 600 mg followed by 300 mg twice daily (starting 12 h after the loading dose), with a total treatment duration of 5 days. An ECG was recorded before the initiation of chloroquine treatment and on a subsequent day of treatment after the first maintenance dose, preferably 24–48 h after the initiation of therapy. For the baseline ECG a time window of a maximum of 24 h before the initiation of chloroquine treatment was required. Chloroquine was not prescribed to patients with a baseline QTc interval duration of >500 ms, in accordance with hospital policy [8]. In patients with a QTc prolongation >500 ms during chloroquine treatment, dose reduction was effectuated, or treatment was stopped.

Data were manually extracted from the hospital information system Epic (Madison, WI, USA). For all ECGs, the heart rate, PR, QRS and QTc intervals were extracted together with the patient’s gender and age. ECGs were standard 12-lead resting ECGs with computerised ECG interpretation. The heart rate, PR interval, QRS interval and QTc interval were computer-interpreted and saved in the hospital information system Epic. The QT interval was adjusted using the Bazett formula to obtain the QTc interval. Additionally, all ECGs were manually interpreted retrospectively by two independent cardiology residents.

The ECG recording during maintenance therapy (at least 12 h after the loading dose) was used to assess the QTc-prolonging potential. If multiple ECGs were recorded during chloroquine therapy, the ECG recording which was closest to the time to reach the maximum plasma concentration (T_max_) of the drug (4–5 h after oral administration) was selected, since chloroquine blocks the hERG channel in a concentration-dependent manner and therefore the maximal blocking effect is expected to be at the T_max_ of the drug [9].

Concurrent use of antiarrhythmic drugs (ATC class C01B) was documented and was defined as at least one administration during chloroquine therapy or within the 24 h before the start of chloroquine therapy. In addition, we captured the site of clinical care at the time of chloroquine initiation (intensive care unit (ICU) or general ward). The patients’ medical records were searched for recordings of presence of pre-existing cardiovascular disease and the baseline serum potassium concentration (i.e. concentration closest to the time of the ECG recording with a maximum time span within 12 h before or after the ECG recording).

Data were analysed with SPSS version 26.0.0 (IBM, New York, USA). Descriptive statistics were used to describe baseline characteristics. The paired samples *t*-test was used to test whether the observed heart rate, PR interval, QRS interval and QTc interval during chloroquine treatment were statistically different from the baseline observations. Multivariate and univariate logistic regression were conducted to search for predictive factors for a marked QTc prolongation (QTc >500 ms) with a significance level of *p* < 0.05.

## Results

A total of 95 patients had both a baseline ECG recording and an ECG recording during chloroquine therapy. Baseline characteristics are displayed in Tab. 1.

**Table 1:** Baseline characteristics

Characteristic	Median (min-max) or number (%)
Age (years)	65 (18–91)
Gender (male)	63 (66%)
Intensive care unit	21 (22%)
Concurrent use of antiarrhythmic drugs	4 (4%)
*Known prior cardiovascular disease*	
– Atrial fibrillation	13 (14%)
– Coronary artery disease	11 (12%)
– Congestive heart failure	9 (9%)
– Other	9 (9%)

The majority (78%) of these patients were admitted to a general ward (e.g. respiratory ward), whereas 22% were admitted to an ICU. Five patients had a baseline QTc interval of at least 500 ms after computerised interpretation which proved to be less than 500 ms after manual review. However, manual interpretation disclosed three patients with a QTc interval of at least 500 ms who originally had a QTc interval of less than 500 ms after computerised interpretation. Of the patients with a baseline QTc of at least 500 ms, none were using antiarrhythmic or other QT-prolonging drugs concurrently. Ten patients (11%) had atrial fibrillation according to the ECG at baseline.

Tab. 2 displays the mean QTc interval before and during chloroquine treatment. Chloroquine treatment resulted in a mean QTc prolongation of 35 ms (95% CI 28–43 ms) using computerised interpretation and 34 ms (95% CI 25–43 ms) on manual interpretation. No torsade de pointes or other clinically relevant ventricular arrhythmias were observed during chloroquine treatment, although patients on the general ward had no rhythm monitoring.

**Table 2:** Effect of chloroquine on QTc interval

	Mean QTc before chloroquine treatment (ms) (95% CI)	Mean QTc during chloroquine treatment (ms) (95% CI)	Mean difference (ms) (95% CI)
Computer interpreted	444 (373–515)	479 (394–564)	35 (28–43)
Manually interpreted	432 (360–505)	466 (383–549)	34 (25–43)

*CI* confidence interval

After manual review, 22 patients (23%) had a QTc interval exceeding 500 ms during chloroquine treatment. None of these patients had a prolonged interval before the initiation of chloroquine therapy and none were using antiarrhythmic drugs concurrently. In all of these patients the chloroquine dose was reduced and, in the case of persistent QTc prolongation, terminated.

**Fig. 1:**
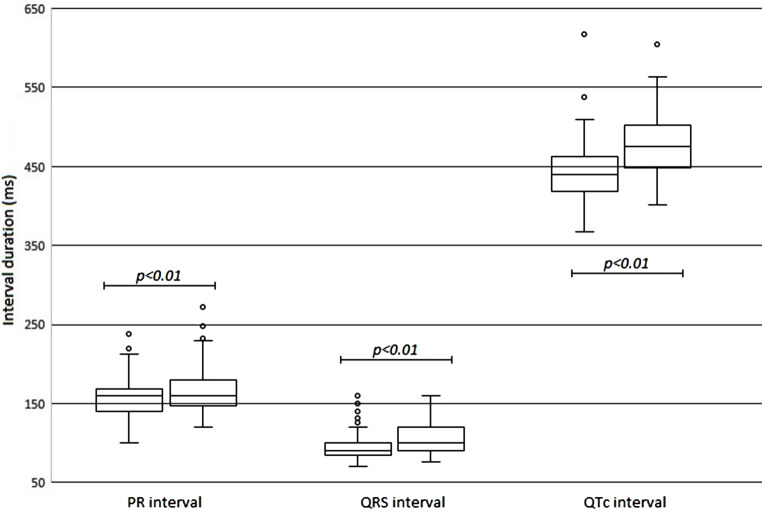
Distribution of PR, QRS and QTc intervals before and during chloroquine treatment after computerised ECG interpretation (*left box* before chloroquine treatment, *right box* during chloroquine treatment)

Statistically significant effects were also observed on heart rate (mean difference −10 bpm), PR interval (mean difference 8 ms) and QRS interval (mean difference 6 ms) (Fig. 1).

In both the univariate and multivariate regression analyses none of the baseline characteristics (see Tab. 1) was associated with having a QTc >500 ms during chloroquine treatment. The mean differences in QTc were 36, 39, 30, 64 and 37 ms for age >70 years, female gender, ICU admission, concurrent use of antiarrhythmic drugs and known prior cardiovascular disease, respectively.

## Discussion

### Main findings

This study shows that treatment of COVID-19 patients with chloroquine resulted in a statistically significant and clinically relevant effect on the QTc interval. In a significant number of patients (23%) chloroquine treatment led to a QTc interval of more than 500 ms, thereby increasing the risk of ventricular arrhythmias. Although the increase in PR and QRS interval duration was statistically significant, this was not considered clinically relevant. The prolongation of the QRS duration influenced the QTc interval, and therefore possibly led to overestimation of the prolongation of the QTc interval.

In the present study, a more pronounced effect on the QTc interval was observed than in other studies. In healthy volunteers receiving a 600-mg dose of chloroquine (*n* = 24), Mzayek et al. observed a mean increase of 16 ms (95% CI 9–23 ms) at 4–5 h post-dose [2]. In a study in healthy Thai volunteers (*n* = 16) a 600-mg dose resulted in a mean QTc interval (Fridericia corrected) prolongation of maximally 6.1 ms [3]. One explanation for the more pronounced QTc prolongation in our study is probably that we measured the interval after multiple doses of chloroquine instead of after a single dose. Since chloroquine has a very large distribution volume (200–800 l/kg), and a long half-life of 20–60 days [10], chloroquine plasma concentrations increase continually during the 5‑day treatment period, since the steady-state concentration has not yet been achieved. Due to concentration-dependent blockade of the hERG channel [5], the effect on the QTc interval is also expected to keep increasing with rising plasma concentrations. Another explanation could be that these patients are severely ill and hence may have ventricular remodelling, which may make them more susceptible.

Since the use of QTc-prolonging drugs is considered to be one of the many risk factors associated with the occurrence of torsade de pointes, other risk factors that are associated with QTc prolongation should be monitored and corrected, such as electrolyte imbalances. In addition, from a pharmacological point of view, concurrent use of other QTc-prolonging drugs should, if possible, be avoided.

### Strengths and limitations

This is a study with a large sample size compared to other studies on this topic and is, to our knowledge, the first to study the QTc-prolonging potential of chloroquine in the context of COVID-19 treatment. A limitation of this study is its retrospective nature with the consequence that ECGs were not captured at exactly the expected time of maximum plasma concentrations. On the other hand, our findings can also be regarded as a good reflection of how ECG monitoring with computerised interpretation takes place in regular practice. In this connection, we would like to mention that the objective of this study was not to study the difference between computerised and manual interpretation of QTc intervals on ECGs or the validity of either method.

## Conclusions

Chloroquine treatment in patients with COVID-19 significantly prolonged the QTc interval by 34–35 ms; 23% of patients had a QTc interval exceeding 500 ms. These findings highlight the need for ECG monitoring when prescribing chloroquine to COVID-19 patients.
